# Methanol Intoxication Presenting With Bilateral Optic Neuritis and Paracentral Acute Middle Maculopathy

**DOI:** 10.7759/cureus.21587

**Published:** 2022-01-25

**Authors:** Sherouk G Saad, Yousef A Fouad, Mohamed Nowara

**Affiliations:** 1 Investigations Unit, Al Mashreq Eye Center, Cairo, EGY; 2 Ophthalmology, Ain Shams University, Cairo, EGY

**Keywords:** toxic optic neuropathy, optic neuritis, optical coherence tomography, paracentral acute middle maculopathy, methanol

## Abstract

A 52-year-old male presented nine days following accidental methanol ingestion with a drop of vision in both eyes. A complete ophthalmic assessment was performed, with a referral to a neurologist for multidisciplinary management. On ophthalmological evaluation, visual acuity was hand motion (HM) bilaterally with swollen optic discs. Macular optical coherence tomography (OCT) showed a picture consistent with paracentral acute middle maculopathy (PAMM) in both eyes, and brain magnetic resonance imaging showed ischemic insults. Slight visual improvement was detected following steroid and antiplatelet therapy. At two months of follow-up, bilateral optic atrophy ensued. Special attention must be given to the macular assessment and not just the optic nerve when considering a case of methanol toxicity, as methanol intoxication can lead to ischemic manifestations.

## Introduction

Methanol is a colorless, toxic alcohol that is used in some industries as solvents, alternative fuel sources, and pesticides and is unfit for human consumption. Toxicity can occur due to accidental or suicidal inhalation, contact, or ingestion, with the latter being the most common type of intoxication [1]. Accidental methanol consumption in alcoholic beverages represents a health burden particularly in developing countries owing to legal constraints and low socioeconomic class [2]. The event can lead to serious morbidity and could even be life-threatening. Reported presentations of methanol intoxication include metabolic acidosis, gastrointestinal manifestations, and serious neurological affection in the form of central nervous system depression that may lead to coma and death [1].

Moreover, ocular toxicity ranging from the blurring of vision to complete blindness is commonly reported [3]. It has been shown that methanol causes a form of toxic optic neuritis, specifically affecting the papillomacular bundle and resulting in bilateral optic disc swelling in the acute stage, followed by optic atrophy later on [4,5].

We herein present a peculiar case of accidental methanol ingestion that presented not only with toxic optic neuropathy but also with paracentral acute middle maculopathy (PAMM).

## Case presentation

**Figure 1:**
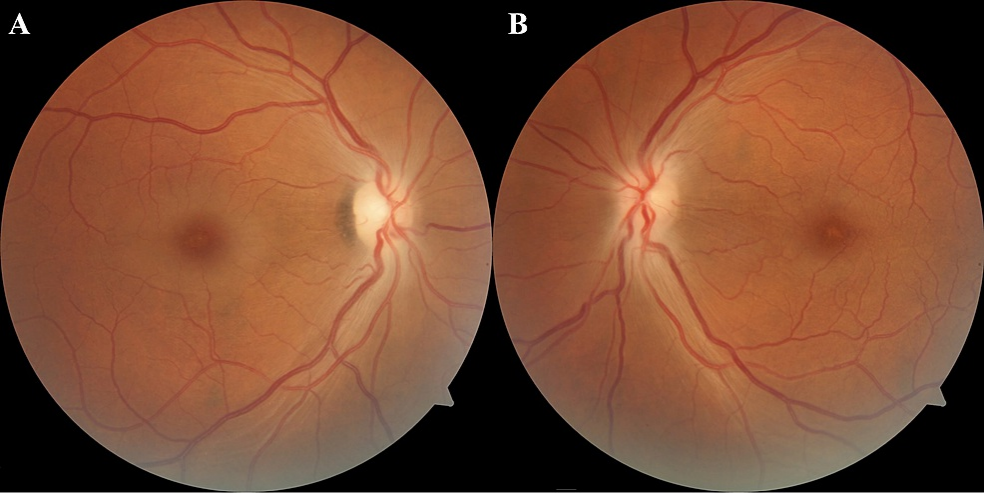
Fundus photographs of the right (A) and left (B) eyes on presentation showing optic nerve edema in both eyes and macular pallor more pronounced in the right eye.

**Figure 2:**
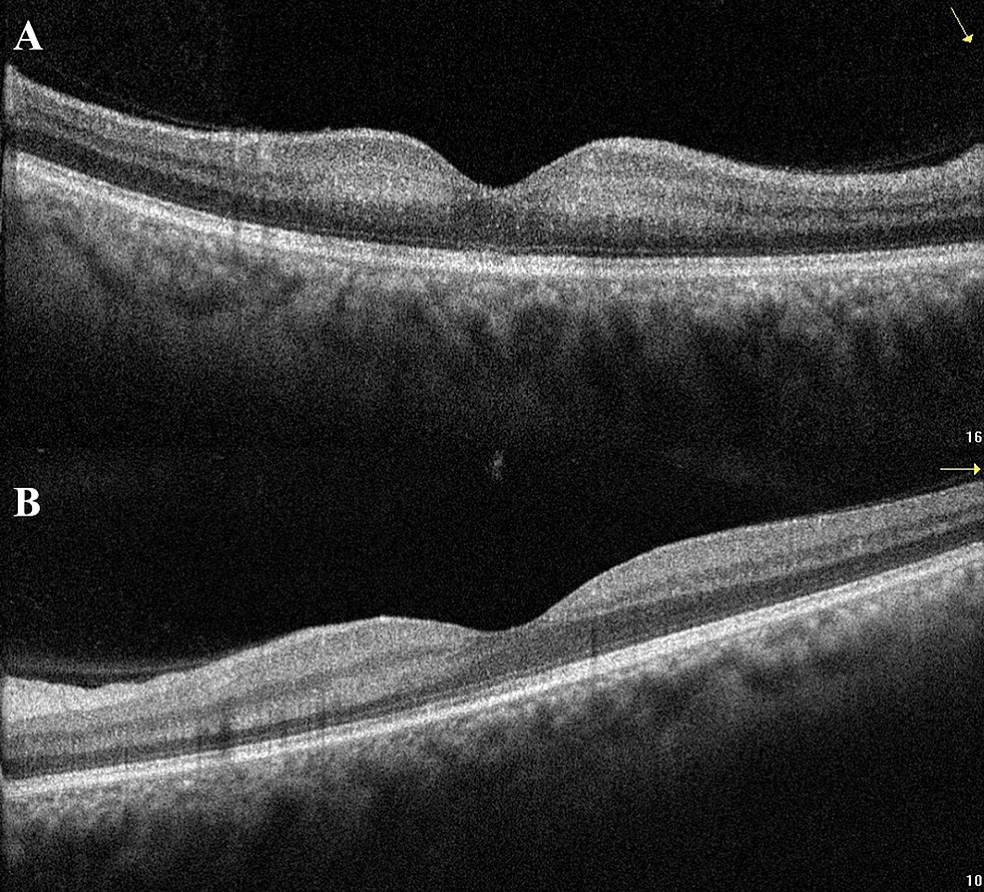
Optical coherence tomography macular line scans of the right (A) and left (B) eyes on presentation, with abnormal hyperreflection at the level of the middle retinal layers sparing the outer layers.

A 52-year-old male with unremarkable medical, ocular, and family history had accidentally ingested methanol alcohol and presented to our care nine days following the incident. He had been suffering from hiccups, severe abdominal pain, and significant sudden vision loss that had started two days following the ingestion. The patient had not received any prior treatment for the condition. On examination, the corrected distance visual acuity (CDVA) in both eyes was hand motion (HM), and his pupils were sluggishly reactive. The intraocular pressure was 19 mmHg in both eyes. Slit-lamp examination of the anterior segment was unremarkable. Fundus examination revealed bilateral optic disc edema with macular pallor more pronounced in the right eye (Figure 1). Optical coherence tomography (OCT) of the macular area showed placoid, hyperreflective bands at the level of the inner nuclear layer, sparing the outer retina, consistent with PAMM (Figure 2).

The patient was referred for a neurological consultation. Contrast-enhanced magnetic resonance imaging of the brain was ordered and showed bilateral symmetrical patchy areas of abnormal signals involving both deep cerebellar regions and both ganglionic and corona radiate areas, bright on T2/fluid-attenuated inversion recovery images, of low signals in T1-weighted scans and restricted in diffuse-weighted imaging with no significant pattern of enhancement. This was consistent with ischemic brain injury. The patient was prescribed oral steroids in the form of methylprednisolone 60 mg per day for one week, proton pump inhibitor 40 mg per day for one week, and intramuscular injections containing vitamin B12. Due to the nature of ischemic injuries, the patient was also prescribed clopidogrel 75 mg once daily.

**Figure 3:**
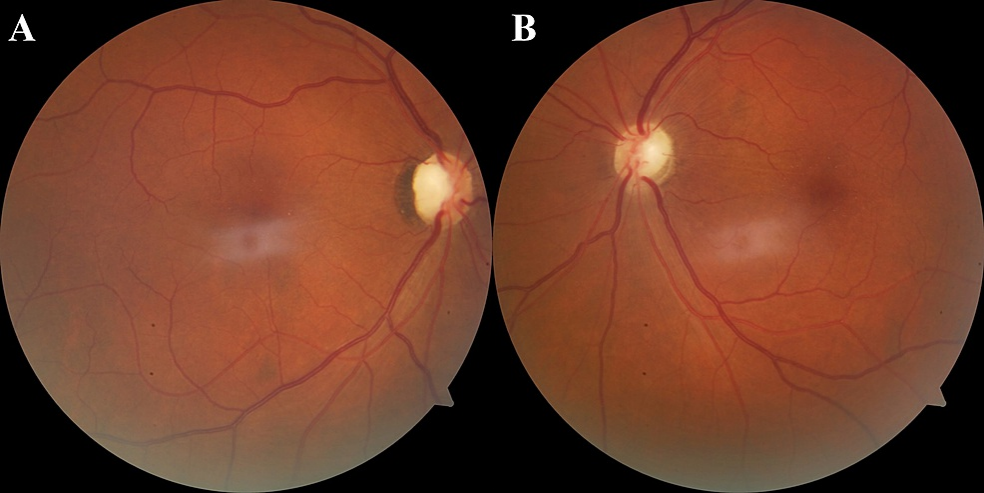
Fundus photographs of the right (A) and left (B) eyes at two months of follow-up demonstrating the ensuing optic atrophy in both eyes.

On the fourth day of treatment, the patient reported improvement of vision in his right eye, and CDVA was counting fingers (CF) at 10 cm and HM in the right and left eyes, respectively. Three days later, the vision continued to improve in both eyes, reaching CF at 50 cm and 10 cm in the right and left eyes, respectively. At that time, steroid tapering was advised. At the final follow-up of the patient at two months, CDVA was CF at 10 and 25 cm in the right and left eyes, respectively. Fundus examination showed bilateral optic atrophy (Figure 3).

## Discussion

Methanol toxicity remains a potentially fatal health burden that is more prevalent in developing countries. It has been well documented that such toxicity causes significant vision loss, decreased color perception, and central, paracentral, and centrocecal scotomas [1,2]. These effects are attributed to formaldehyde and formic acid (oxidative end products of methanol) causing adenosine triphosphate deficiency resulting in severe tissue hypoxia and retinal nerve fiber layer demyelination [6]. Other unusual ocular presentations have been reported with methanol intoxication. For example, a case in which bilateral serous retinal pigment epithelium detachments occurred in association with optic neuritis was reported [7].

PAMM is an OCT finding that may be isolated or associated with other retinal diseases. Although the exact pathophysiology of PAMM is not fully understood, it is thought to be caused by ischemia in the deep or intermediate capillary plexuses [8,9]. Other modalities useful for detecting PAMM include OCT angiography, which shows variable areas of capillary dropout, and en face OCT, which shows confluent areas of middle retinal hyperreflectivity with three distinct patterns: arteriolar, fernlike, and globular [10]. However, the superiority of one modality over the other in aiding PAMM diagnosis has not been proven. Limitations to our report include the lack of en face and OCT angiography imaging and the unavailability of follow-up OCT scans. The most important differential of PAMM is acute macular neuroretinopathy, which, unlike PAMM, on OCT shows hyperreflectivity at the junction of the outer plexiform layer and the outer nuclear layer and may be associated with disruption of the ellipsoid and interdigitation zones [11].

Pattern electrophysiological studies in cases with methanol intoxication have demonstrated a decrease in amplitude and number of oscillatory potentials, in addition to a decrease in response with a delayed peak of b-wave; this suggests an affection of bipolar and Muller cells [6]. It is unclear whether PAMM occurrence in this case is related to direct ischemia of the inner retinal layers, oxidative damage, or a mixture of both. The occurrence of ischemic brain insult suggests a circulatory defect caused by intoxication or possibly other impurities.

## Conclusions

Ocular methanol toxicity not only involves the optic nerve but may also result in a retinal vascular insult. This highlights the value of multimodal ocular investigation in cases of methanol intoxication, especially when there is suspicion on fundus examination.
